# HOMA estimated insulin resistance as a marker for angiographic severity of coronary artery disease in non-diabetic and non-obese patients

**DOI:** 10.22088/cjim.14.3.495

**Published:** 2023

**Authors:** Mohamed Eid, Sherif A. Sayed, Nayel A. Zaki, Amera M. F. Hamdy, Ali M. A. Altaher

**Affiliations:** 1 Department of Internal Medicine, Faculty of Medicine, Sohag University, Sohag, Egypt; 2 Department of Clinical Pathology, Faculty of Medicine, Sohag University, Sohag, Egypt; 3 Department of Medical Biochemistry, Faculty of Medicine, Sohag University, Sohag, Egypt

**Keywords:** Coronary artery disease, Coronary atherosclerosis, HOMA estimated insulin resistance.

## Abstract

**Background:**

Insulin resistance (IR) examined by homeostasis model assessment of insulin resistance (HOMA-IR) measures increased carotid atherosclerosis risks in non-diabetic and non-obese persons. The information about the relationship between IR and coronary atherosclerosis severity among non-diabetic and non- obese population showed conflicting results. The goal of the current study was to assess the relationship that links IR and coronary artery disease (CAD) among non-diabetic and non-obese Egyptian populations.

**Methods:**

112 non-diabetic and non-obese patients were included in this study. These patients underwent coronary angiogram (CA) either due to acute coronary syndrome (ACS) or chronic stable angina with positive exercise tests.

**Results:**

Our study found a strong association between HOMA-IR and Gensini score (r = 0.831, with p<0.001) in general population. The HOMA-IR was the most influential independent predictor for the presence of extensive coronary atherosclerosis. Non-diabetic and non-obese people carrying the risk of developing the three-vessel disease (3VD) may be identified with a HOMA-IR score of over 5.9 according to the ROC curve analysis with a sensitivity of 67.7%.

**Conclusion:**

IR that developes in non-diabetic non-obese individuals coincides with more severe and more outspread CAD. In non-diabetic non-obese populations who need a CA, a single HOMA-IR value is higher than 5.9 may imply an elevated risk for clinically significant CAD. As insulin resistance is a reversible process, risk stratifications of CAD in non-diabetic non-obese populations could include HOMA-IR as one of its parameters.

Type 2 diabetes mellitus (T2DM) is responsible for majority of mortality reasons worldwide owing to its high prevalence and doubling the risk of heart diseases (1). Rising glucose and insulin levels are associated with heart disease risk (2, 3). It is hard to assess the involvement of insulin resistance (IR) in the prognosis of coronary artery disease (CAD) among diabetic patients as all T2DM patients are insulin resistant. Both IR and hyperglycemia play key roles in the initial stages of atherosclerosis, as shown in the in vivo studies (4). IR which is the pathological hallmark for the development of T2DM, may occur before T2DM clinical diagnosis in many years, besides that, hyperinsulinemia that occurs before abnormal glucose levels shows several pathophysiological abnormalities which exert deleterious effects (5, 6). Therefore, many researches have been proposed that the development of T2DM goes through three stages: stage of hyperinsulinemia, stage of prediabetes, and lastly, stage of diabetes (7).

Previous studies found that IR occurs even early in physiological states. Reaven et al. demonstrated that IR measured by glucose clamp technique is present in about 25% of non-obese individuals having a normal oral glucose tolerance (8). Also, Pyörälä et al. scrutinized the relevance of IR in apparently normal people. This was assessed in about 970 healthy men and follow-up extended up to 22 years and proved the rise of the risk of coronary atherosclerosis (9). The link between insulin resistance and obesity is studied well in previous literature (10), however there are multiple researchers found that persons with normal body weight have IR too and, if left uncorrected, may ultimately lead to development of T2DM and consequently possible cardiovascular complications although they were not obese (11). Non-alcoholic fatty liver disease (NAFLD) is proven to be a distinct risk to IR in obese individuals and those with metabolic syndrome. However,NAFLD and IR can also occur in overweight or lean people (12). Moreover, De fronzo et al. found that people having a body mass index (BMI) between (20 – 27) kg/m^2^ who gained more by 2 to 10 kg fat during their adult stage comprises ideal candidates to acquire IR and thereafter T2DM (13).

The action of insulin on the arterial wall appears to be altered by IR; in the insulin-sensitive condition, insulin has an anti-atherogenic effect, while in the IR condition, the opposite is true (14). Also, IR in type 1 diabetes mellitus is responsible of more CAD risk. (15, 16). Homeostasis model assessment of insulin resistance (HOMA-IR) is a measure that incorporates fasting blood glucose (FBG) and fasting insulin levels. It is considered a credible indication of IR as it has been proven to correlate well with the hyperinsulinemia-euglycemic clamp (17). High insulin level is deleterious in people with abnormal or even normal glucose tolerance. Two previous scholars (18, 19) demonstrated the deleterious effect of fasting hyperinsulinemia or post oral glucose load on CAD events was independent of different conventional CAD risk factors such as dyslipidemia, smoking, hypertension, physical status and obesity indices.

In participants with normal levels of FBG and glucose tolerance, IR increased carotid atherosclerosis risks (20). Also, high HOMA-IR was linked to severe coronary atherosclerosis >50% in non-obese non-T2DM individuals, as demonstrated by Mossmann *et al.* (2015) (21). The relation CAD and IR may be distinct from clinical risk factors (22). It has been notarized that, hyperinsulinemia can predict coronary calcification progression documented by coronary calcium score in apparently healthy individuals with ages (60–72) years after 24 months follow-up. This was independently evidenced from risk factors such as diabetes mellitus, hypertension, dyslipidemia and ethnicity, concluding that IR is a powerful distinct predictor of CAD progression (23). IR and concurrent hyperinsulinemia exist long before pre diabetes or in the development of frank T2DM, so if an intervention treatment can be applied during the pre-diabetes stage or even early in the stage of hyperinsulinemia, we can inhibit or slow the evolution of T2DM and its subsequent hazardous complications. So a question rises here, if IR in non-diabetic and non-obese individuals with normal levels of fasting blood glucose can promote coronary atherosclerosis and to what extent? In the present study, our goal was to study the relation between the HOMA-IR level and the severity of coronary atherosclerosis in non-diabetic and non-obese populations’ candidate for coronary angiogram.

## Methods

**Patients: **This was a prospective observational study carried out in the Cathlab Unit of Internal Medicine Department, Faculty of Medicine, Sohag University from October 2020 to October 2021. We analyzed consecutive patient candidates for coronary angiogram (CA) either due to chronic stable angina or acute coronary syndrome (ACS). For patients with chronic stable angina, CA was indicated in the presence of the following criteria:

(1) Clinical likelihood of CAD is high and unresponsive symptoms to medical therapy.

(2) Typical symptoms of angina at a low exercise level without previous non-invasive risk stratifications.

(3) Symptoms of angina which were not elucidated enough by non-invasive measures. In the absence of the previous criteria, CA was indicated after the documentation of positive myocardial ischemia by non-invasive exercise tests (24). 

Patients aged less than 18 years, pre-diabetes, diabetes and BMI >30 Kg/m^2^, pregnant or lactating women, anemia, chronic kidney disease, associated valvular heart disease, heart failure were excluded. We used FBG from 100 to <126 mg/dL or the glycosylated hemoglobin (HbA1c) from 5.6 to < 6.4% to define pre-diabetes and FBG equal to or more than 126 mg/dL and Hb1Ac level of equal to or more than 6.5% for overt diabetes diagnosis (25). This work was approved by Ethics Board of Sohag University with IRB registration number Soh-Med-22-01-42 with reference to the Helsinki Declaration guidelines for human research studies available at https://www.wma.net/. An informed written consent was taken from each participant. All subjects were assessed by history taking, clinical examination, and laboratory investigation. The data about personal history as age, sex, smoking status, and positive family history of premature CAD was collected. 

Laboratory investigation included serum total cholesterol (TC), low-density lipoprotein cholesterol (LDL), triglyceride (TG), fasting glucose, fasting insulin, serum creatinine and estimated glomerular filtration rate (e-GFR). Complete blood count (CBC) was done by CELL-DYN 3700 (Abbott Laboratories, diagnostic division IL, USA). Serum creatinine, TC, TG, LDL were performed by Cobas C 311 chemistry analyzer system (Roche Diagnostic, G mbH Indianapolis, IN, USA). HbA1c test was performed by architect i000 SR system (Abbott Laboratories, diagnostic division IL, USA). Resting 12 lead surface electrocardiography was done to all patients to determine the signs of ischemia by using Japanese Fukuda device (Cardimax FX 8200). Blood pressure (BP) values of ≥140/90 mmHg, and/or on current therapy for hypertension was used to define hypertension (26). Positive family history of premature CAD was deemed present if a person had first-degree relatives with history of CAD or sudden death before the age of 65 and 55 for women and men, respectively. BMI was calculated by dividing the weight in kilograms by square of body height in meters. Obesity was defined if BMI was >30 or more (27). Total serum cholesterol levels above 200 mg/dL were considered hypercholesterolemic (28). Fasting TG level above 150 mg/dL was considered elevated. Hypercholesterolemia and/or hypertriglyceridemia or if patient had already been taking lipid lowering therapy were categorized as dyslipidemia. (28). 

**Coronary atherosclerosis assessment of the:** Selective CA was performed via femoral approach using Seldinger's method (29). By the use of cine-angiographic equipment (Toshiba. Biplane Infinix CB, Japan), multiple observations were recorded from different views. The angiogram analysis was done by two experienced intervention cardiologists, both not related to the research area. Gensini score and the number of vessels with significant stenosis (vessel score) were used to assess the severity of CAD (30). Vessel score is the number of vessels having significant stenotic lesions, which is the vessel number with at least a 50% decrease in the luminal diameter of one of coronary arteries. The coronary tree including left main coronary artery (LM) and right coronary artery (RCA), LM artery then divides into left anterior descending artery (LAD) and left circumflex artery (LCX). 

The score ranged from 0 to 3, which were dependent on the vessel(s) involved. Gensini score, described previously, gave a 1 for 25% stenosis and 2 for 50%, 4 for 75%, 8 for 90%, 16 for 99%, and 32 for the total occlusion. The scores were then multiplied by a factor based on the significance of the lesion's location in the coronary artery. LM lesion had a score of 5, proximal LAD and proximal LCX had a score of 2.5, middle portion of LAD had a score of 1.5, distal LAD, the middle and distal segments of LCX, and RCA had a score of 1, while a score of 0.5 was given for other branches as diagonal artery, obtuse marginal artery. The sum of all sector scores hand out the Gensini score, which emphasizes the degree of disease severity.

**Blood assay:** At the time of enrolment in the trial, all participants had their venous blood drawn following an overnight fast (water was allowed) for 12 hours in stable hemodynamic conditions. The glucokinase technique was used to test plasma glucose. The Tosoh AIA-360 automated immunoassay analyzer was used to measure the insulin level. It is a two-step chemiluminescence enzyme immunoassay (CLEIA). The brochure claims that it is done in a specific cup. The anti-insulin mouse monoclonal antibody mounted on magnetic microparticles in one cell binds insulin contained in a test sample (Cell-I). A precise amount of enzyme-labeled anti-insulin mouse monoclonal antibody reconstituted in another cell (Cell-II) was poured into cell-I after the magnetic micro-particles were first incubated to eliminate unattached materials. 

To eliminate unbound enzyme-labeled monoclonal antibodies, the magnetic microparticles were rewashed and treated with a chemiluminescent substrate. The insulin level was closely related to the quantity of enzyme-labeled monoclonal antibodies that bind to magnetic microparticles. According to the HOMA-IR model, an estimated IR was obtained according to this equation: fasting insulin (mU/L) × fasting glucose (mmol/L) / 22.5. Pathology was declared when the HOMA-IR values fell below the 75^th^ percentile (31).

**Analysis of the statistics:** We used the Statistical Package for Social Sciences (SPSS) Version 22 (SPSS Inc, Chicago, USA) for analysis. A *p-value* < 0.05 was considered significant. The student t-test, Mann-Whitney, chi-square, Friedman’s test, ROC curve, and *Spearman's* correlation were adopted.

## Results

A total of 112 participants out of 300 who had CA for ACS or SCAD were engaged in our analysis, while we excluded 188 because of being pre-diabetics or diabetics or obese (BMI≥ 30kg/m^2^). Males were 75 (67%) and 37 (33%) were females. The mean patient age was 61.08±6.81 (44.0–77.0) years. Results of HOMA-IR testing separated patients into two distinct groups; group A [HOMA IR <2.5 considered insulin sensitive] & group B [HOMA IR ≥ 2.5 considered insulin resistant]. An example of the diagnostic CA is shown in figuer 1. 

**Figure 1:**
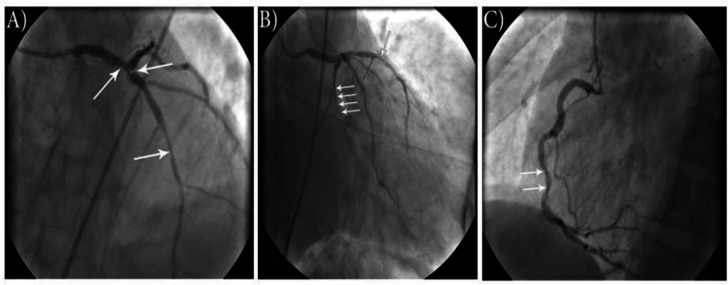
A coronary angiogram of a non-diabetic male (54 years old) with high HOMA-IR who showed three-vessel disease (white arrows) and a high Gensini score. A: distal LM artery stenosis (80%), proximal LAD stenosis (99%) & mid LAD stenosis (90%). Cranial view, B: proximal LCX total occlusion (100%) & mid LAD stenosis (90%). Caudal view, C: mid RCA stenosis (70%).

Although not reaching a significant statistical difference, BMI and TG were higher in group (B); the mean BMI in group (A) was 25.63± 1.83 and 26.16± 1.74 in group B with a p- value 0.06 while the mean TG level was 121.43± 47.91 in group A and 131.31± 40.76 in group B with P=0.057.The demographic features of the participants in the two groups are given in table 1. Both groups were comparable, considering age, gender, risk factors, also regarding the clinical data and laboratory investigations. There was no significant difference in both groups apart from LDL, which showed significant elevation in group B (P=0.048). 

**Figure 2:**
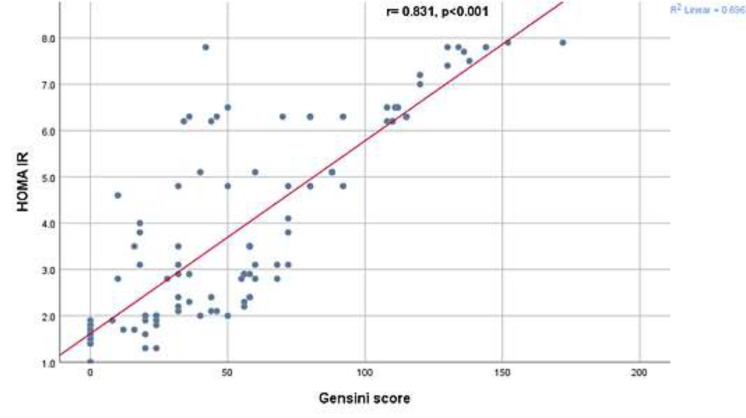
Correlation between the Gensini score and HOMA IR of the study patients

**Figure 3:**
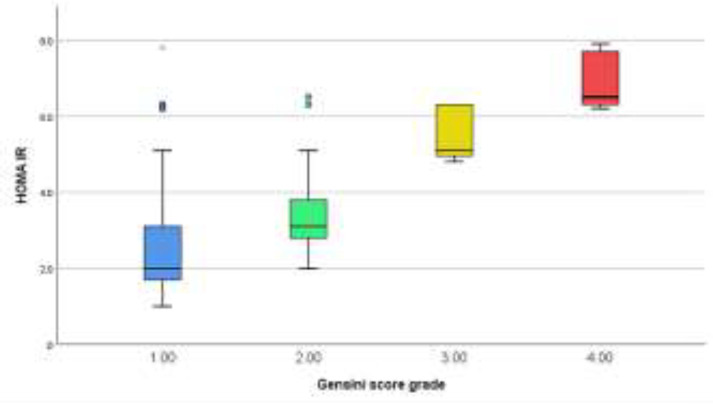
Relation between the HOMA-IR and CAD severity according to Gensini score of the study patients

**Figure 4.**
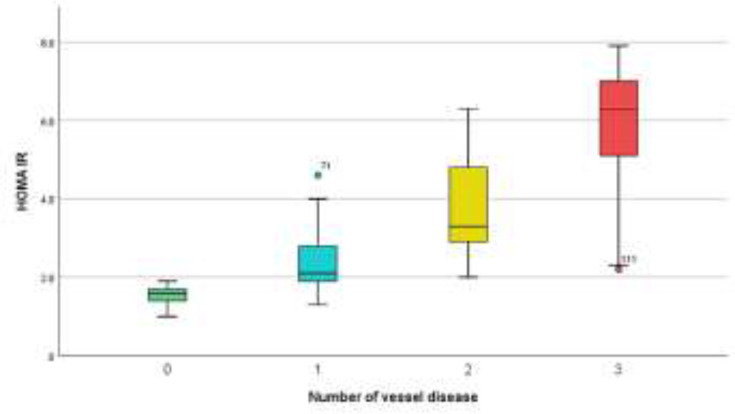
Relation between the HOMA-IR and CAD extent according to the number of vessel disease of the study patients

Regarding diagnosis, group A had 17 (42.5%), and Group B had 29 (40.3%) patients with ACS. Group A had 23 (57.5%), and Group B had 43 (59.7%) patients with stable CAD (SCAD), with no significant difference (P= 0.819), as shown in table 2. When the HOMA-IR, number of diseased vessels and Gensini score were studied, they showed statistically significant elevation in group (B), (P<0.001), as shown in table 3. The HOMA-IR and Gensini scores had a significant correlation (r = 0.831, with P<0.001) in general population (Figure 2). Women had a little better association than males (r = 0.835, with P<0.001 vs. r = 0.824, with P<0.001). Apart from BMI and dyslipidemia, there was no correlation between the HOMA-IR and Gensini score based on any of the pre-existing baseline characteristics (sex, age, smoking, hypertension, and family history). HOMA-IR was shown to rise in conjunction with a rise in the Gensini score and extension of coronary atherosclerosis from one to three vessels (Figures 3&4). 

**Figure 5:**
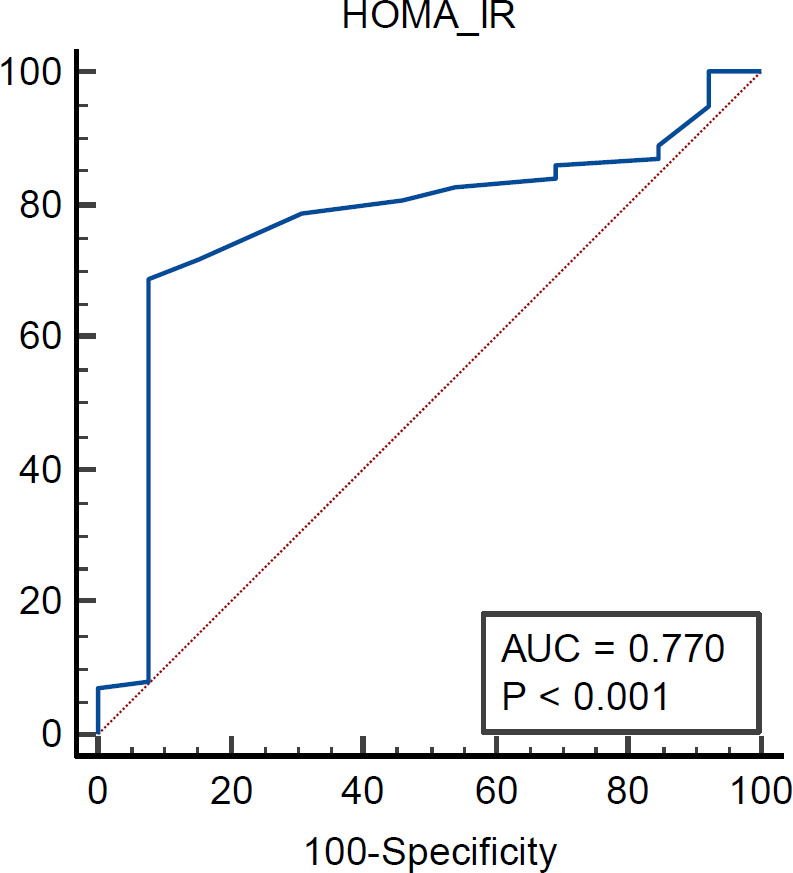
ROC curve for HOMA-IR in the prediction of 3VD area under curve [AUC] is 0.77. HOMA IR has a good predicting validity in patients with or without 3VD

We found that HOMA-IR was powerful foreteller of severe (Gensini score grade 4) coronary atherosclerosis, and also it was independent and strongest predictor for three-vessel disease CAD (OR= 2.954, P< 0.001) as evidenced by binary logistic regression analysis (tables 4 & 5). Using the receiver operating characteristic (ROC) curve analysis illustrated that individuals without diabetes or obesity who were at elevated risk of having three-vessel disease had a HOMA-IR score higher than 5.9 with 67% sensitivity and 92.3% specificity (AUC= 0.770, P<0.001) (table 6 & figure 5). 

**Table 1:** Clinical features, investigation data and risk factors of included patients

		**Group (A)** **(HOMA IR ≤2.5)** **(40)**		**Group (B)** **(HOMA IR >2.5)** **(72)**		**P- value**
		**N**	**%**	**N**	**%**	
**Age (years)**	mean± SD median (IQR) Range	61.25± 6.67 61.0 (59.0- 65.0) 48.0- 73.0		60.99± 6.93 61.0 (59.0-64.50) 44.0- 77.0		0.927^╪^
**Sex**	Male	27	67.5%	48	66.7%	0.928^‡^
	Female	13	32.5%	24	33.3%	
**Risk factors**	Hypertension	12	30.0%	24	33.3%	0.717^‡^
	Smoking	14	35.0%	24	33.3%	0.858^‡^
	Dyslipidemia	13	32.5%	24	33.3%	0.928^‡^
	Family history of CAD	6	15.0%	12	16.7%	0.818^‡^
**BMI (Kg/m** ^2^ **)**	Mean± SD median (IQR) Range	25.63± 1.83 25.45 (24.0-26.30) 23.0- 29.11		26.16± 1.74 26.0 (25.0- 27.45) 23.40- 29.90		0.067^╪^
**SBP (mm/Hg)**	Mean± SD median (IQR) Range	129.87± 12.0 128.5 (123.0-135.5) 105.0- 160.0		133.63± 11.27 135.0 (124.5-140.0) 109.0- 170.0		0.102^*^
**DBP (mm/Hg)**	Mean± SD median (IQR) Range	76.60± 8.29 74.50 (70.0-83.50) 64.0- 92.0		77.56± 7.74 77.0 (70.0-83.50) 65.0- 98.0		0.467^╪^
**TC (mg/dl)**	Mean± SD median (IQR) Range	150.05± 47.78 133.0 (109.5- 201.0) 80.0- 231.0		159.0± 45.73 150.5 (114.5- 201.0) 88.0- 235.0		0.255^╪^
**TG (mg/dl)**	Mean± SD median (IQR) Range	121.43± 47.91 100.0 (79.0- 178.0) 70.0- 200.0		131.31± 40.76 120.5 (98.5- 173.5) 78.0- 210.0		0.057^╪^
**LDL (mg/dl)**	Mean± SD median (IQR) Range	84.33± 23.81 77.50 (60.0- 101.50) 59.0- 126.0		90.50± 19.45 87.50 (71.0- 100.0) 66.0- 132.0		0.048^╪^

‡ Chi-square test, * Student T test, ╪ Mann- Whitney U test, SD: standard deviation, N: patients' number

CAD: coronary artery disease, TC: serum total cholesterol, TG: serum triglyceride, BMI: body mass index, SBP: systolic blood pressure, DBP: diastolic blood pressure, LDL: low density lipoprotein.

**Table 2.** Presentation characteristics in both groups

		**Group (A)** **(HOMA IR ≤2.5)** **(40)**		**Group (B)** **(HOMA IR>2.5)** **(72)**		**P- value**
		**N**	**%**	**N**	**%**	
**Diagnosis**	**ACS**	17	42.5%	29	40.3%	0.819^‡^
	**SCAD**	23	57.5%	43	59.7%	

N: patients' number, ACS: acute coronary syndrome, SCAD: stable CAD, ‡ Chi-square test.

**Table 3:** HOMA-IR, Gensini score, number of vessel disease characteristics in the two studied groups

		**Group (A)** **(HOMA IR ≤2.5)** **(40)**		**Group (B)** **(HOMA IR >2.5)** **(72)**			**P-value**
		**N**	**%**	**N**		**%**	
**HOMA-IR**	Mean± SD median Range	1.85± 0.37 1.90 (1.60- 2.10) 1.0- 2.40			5.14± 1.68 5.10 (3.50- 6.30) 2.80- 7.90		<0.001^╪^
**Gensini ** **score**	Mean± SD median Range	21.60± 19.42 20.0 (0.0- 34.0) 0.0- 58.0			75.88± 38.28 72.0 (48.0- 110.0) 10.0- 172.0		<0.001^╪^
**Vessel score**	No VD	13	32.5%	0		0.0%	<0.001^‡^
	1 VD	18	45.0%	11		15.3%	
	2 VD	5	12.5%	23		31.9%	
	3 VD	4	10.0%	38		52.8%	

HOMA-IR: Homeostasis model assessment of insulin resistance, ‡ Chi-square test, ╪ Mann- Whitney U test, SD: standard deviation, VD: vessel disease

**Table 4:** The adjusted binary logistic regression analysis for the highest point of Gensini score

**Parameters**	**B**	**S.E.**	**Wald**	**P-value**	**Odds ratio (OR)**	**95% CI**	
						**Lower limit**	**Upper limit**
**Age**	-.043-	.035	1.553	.213	.958	.895	1.025
**Sex**	-.629-	.554	1.288	.256	.533	.180	1.580
**BMI**	1.089	.286	14.504	<0.001	2.970	1.696	5.202
**TC**	.031	.019	2.773	.096	1.032	.994	1.071
**TG**	.026	.017	2.521	.112	1.027	.994	1.061
**LDL**	-.007-	.028	.073	.787	.993	.940	1.048
**SBP**	.087	.045	3.635	.057	1.090	.998	1.192
**DBP**	.002	.059	.002	.968	1.002	.893	1.125
**Hypertension**	.909	.595	2.336	.126	2.483	.773	7.969
**Smoking**	.908	.596	2.337	.127	2.483	.773	7.999
**Dyslipidemia**	-1.138-	.489	5.423	.020	.321	.123	.835
**Family history of CAD**	-.188-	.625	.090	.764	.829	.244	2.820
**HOMA-IR**	2.251	.565	15.849	<0.001	2.496	1.135	5.763

CI: Confidence interval, S.E.: Standard error, B: Regression coefficient; BMI: body mass index, TC: serum total cholesterol, TG: serum triglyceride, DBP: diastolic blood pressure, SBP: systolic blood pressure LDL: low density lipoprotein, CAD, coronary artery disease, HOMA-IR: Homeostasis model assessment of insulin resistance.

**Table 5:** The adjusted binary logistic regression analysis for three-vessel disease

**Parameters**	**B**	**S.E.**	**Wald**	**P-value**	**Odds ratio (OR)**	**95% CI**	
						**Lower limit**	**Upper limit**
**Age**	-.016-	.047	.112	.738	.985	.899	1.079
**Sex**	-.118-	.638	.034	.853	.889	.255	3.102
**BMI**	.234	.201	1.362	.243	1.264	.853	1.874
**TC**	.008	.013	.366	.545	1.008	.983	1.034
**TG**	.001	.014	.003	.959	1.001	.974	1.029
**LDL**	-.030-	.026	1.331	.249	.971	.923	1.021
**SBP**	.037	.040	.861	.353	1.038	.959	1.123
**DBP**	-.029-	.057	.261	.609	.971	.869	1.086
**Hypertension**	.951	.629	2.283	.131	2.588	.754	8.887
**Smoking**	.995	.892	1.243	.265	2.704	.471	15.541
**Dyslipidemia**	.986	.902	1.197	.274	2.682	.458	15.704
**Family history of CAD**	.027	.762	.001	.972	1.027	.231	4.578
**HOMA-IR**	1.600	.477	11.231	.001	2.954	1.943	3.630

CI: Confidence interval, S.E.: Standard error, B: Regression coefficient; BMI: body mass index, TC: serum total cholesterol, TG: serum triglyceride, DBP: diastolic blood pressure, SBP: systolic blood pressure, LDL: low density lipoprotein, CAD, coronary artery disease, HOMA-IR: Homeostasis model assessment of insulin resistance.

**Table 6.** Validity of HOMA-IR in prediction of three-vessel disease

**parameters**	**Best Cutoff value**	**(AUC)**	**(Sensitivity)**	**(Specificity)**	**(PPV)**	**(NPV)**	**P- value**
**HOMA-IR**	>5.9	0.770	68.7%	92.3%	89.9%	74.7%	<0.001

## Discussion

Cardiovascular disease (CVD) is contributing to majority of worldwide mortality. Comorbidities include obesity, altered lipid profiles, and IR often linked to CVD. IR induces several metabolic changes that may lead to heart disease. An imbalance in glucose metabolism caused by IR may result in permanent hyperglycemia, which in turn produces inflammation and increased oxidative stress that results in cell damage. Myocardial IR damages the heart by at least three mechanism: (a) change in signal transduction, (b) poor substrates metabolism control, and (c) modified substrates transport (32). Hyperinsulinemic clamp (HIC) has long been considered the gold standard method for assessing IR, although being time-consuming, costly and hard to use in clinical settings. The HOMA model was used in this work as a surrogate measure of IR because of being simple, practical, and affordable.

For individuals without diabetes and obesity who underwent coronary angiogram, we identified a strong correlation between elevated level of HOMA IR (above the 75^th^ percentile) and the existence of severe coronary atherosclerosis. Non-diabetic, non-obese populations at risk of developing the three-vessel disease may be identified by a HOMA-IR over 5.9 (67.7% sensitivity and 92.3% specificity). These findings are harmonious with a previous study that found a relation between HOMA-IR and CAD in both T2DM and non-DM individuals. A HOMA-IR value more than or equal to 6.0 was shown to be an accurate predictor of severe CAD in that research paper (PPV 82.3%). The most notable finding was considering HOMA-IR as a fundamental predictor of increasing risk for CVD in nondiabetic populations (33). Hedblad et al., (34) conducted a large observational research paper. They examined and divided normoglycemic people without a history of cardiovascular disease into 2 groups according to the 75^th^ percentile of the HOMA-IR test's results: With and without IR. They discovered that CVD risk and mortality was more than double in people with HOMA-IR levels higher than the p 75. Also, IR levels were found to increase and linked to more severe stroke in patients without diabetes (35).

It is worth noting that the HOMA and Gensini score correlation were stronger in females than in males, which might be due to the loss of sex hormones protective effects after menopause, given that average age in our samples was 61.08 +6.81 years. The results of Os et al., (36) support this concept, showing that short duration treatment with estradiol decreased IR in non-diabetic females having CVD. It was demonstrated by Kim et al., that age increased IR (37). IR is assumed to increase CVD risk. When IR is present, the endothelium's ability to inhibit inflammation and sclerosis by secreting NO is compromised. There are fewer receptors, and the phosphoinositide 3-kinase pathway has changed (38).

A few researches studied the relation between vascular atherosclerosis and IR. According to Karrowni et al., (39) non-diabetic individuals after myocardial infarction who had undergone CA had an independent connection between IR and multi-vessel CAD. Srinivasan et al., (40) investigated 61 T2DM patients with CA. The coronary atherosclerosis severity, as determined by Gensini score, was linked positively with the log-HOMA-IR. HOMA-IR was shown to be the best independent predictor of severe and widespread coronary atherosclerosis in a recent research paper by Strisciuglio et al., (41). The current work found that HOMA-IR was the most significant independent foreteller of severe CAD (P=0.001). Previous literatures documented the association between obesity and IR (10), however many studies have shown that IR can also develop in individuals with normal body weight (11). In our study, most of patients with IR were overweight ( mean±SD was 26.16± 1.74) which could also clarify the association between increasing BMI and wide spread coronary atherosclerosis along with IR. It is well established that the 3VD prevalence is higher in patients with T2DM compared to patients without diabetes. But few studies investigated this prevalence in non-diabetic patients with normal HbA1c but not excluding obese patients (41), other researchers studied the link between IR and how many vessels had stenotic lesions at CA in patients without T2DM and obesity but not excluding pre diabetic patients (21). For the best of our knowledge, the link between IR and the extent of coronary atherosclerosis in non-diabetic or prediabetic patients and non-obese patients (as we excluded patients with BMI>30 kg\m2) has never been investigated before. IR is not an irreversible state. Many factors may predispose to it such as dietary habits, physical inactivity and central fat distribution and these factors are potentially reversible. Thus, identification of IR indiviuals with normal glycemia could select high risk patients who are potentially beneficiary from specific interventions either medications, behavioral or dietary. This is demonstrated in a study carried out by De lima Sanches and his colleagues. They found that losing weight encouraged a lowering in IMT of common carotid artery in populations with obesity and this amelioration was mostly due to IR reduction (42). In contrary to our results, some studies documented lack of a positive association between HOMA- IR and CAD angiographic severity (1, 43, 44). Differences in other factors that interact in the pathogenesis of CAD between countries like dietary habits, physical activity, genetic factors and stress conditions may explain these contradictory results and large multicenter trials are needed for more elucidation of this relation. Egypt is a country that has particular features that augment the problem of IR even in non-diabetic and non- obese population. The Egyptian diet is characterized by foods with elevated glycemic index, specially polished rice and white bread. Also Egypt has one of the highest consumption of trans fat worldwide. This very unhealthy type of fat has been found to increase IR and CAD. Morevere, physical inactivity and increasing weight specially visceral adiposity are common risk factors for IR and CAD in Egypt that make about of 60-80% of Egyptian women and half of men overweight or obese (45). Besides, Egypt is ranked the 5^th^ country in Africa in consuming pesticides and a rising proof hints a strong association between the increased risk of IR and exposure to these pesticides (46). All of these factors may explain partially the abovementioned contradictory results. Our research paper was limited by a small but homogeneous sample of individuals undergoing CA. Many patients were already taking drugs for other CAD risk factors, which may have obscured the link between IR and CAD extensions. Also fasting glucose and insulin serum values in ACS individuals may be somehow influenced by the acute event. We may deduce that IR in non-diabetic non-obese individuals goes parallel with CAD severity and expansions. In those patients who need CA, a single HOMA-IR value higher than 5.9 may implies an elevated risk for clinically significant CAD. As insulin resistance is not an irreversible condition, risk stratifications of CVD in patients who are non-diabetic or non-obese could include HOMA-IR as one of its parameters.

## References

[1] Vafaeimanesh J, Parham M, Norouzi S, Hamednasimi P, Bagherzadeh M (2018). Insulin resistance and coronary artery disease in non-diabetic patients: Is there any correlation?. Caspian J Intern Med.

[2] Oguntibeju OO (2019). Type 2 diabetes mellitus, oxidative stress and inflammation: examining the links. Int J Physiol Pathophysiol Pharmacol.

[3] Yu Q, Gao F, Ma XL (2011). Insulin says NO to cardiovascular disease. Cardiovasc Res.

[4] Bornfeldt KE, Tabas I (2011). Insulin resistance, hyperglycemia, and atherosclerosis. Cell Metab.

[5] Haffner SM, Stern MP, Hazuda HP, Mitchell BD, Patterson JK (1990). Cardiovascular risk factors in confirmed prediabetic individuals Does the clock for coronary heart disease start ticking before the onset of clinical diabetes?. JAMA.

[6] Harris MI, Klein R, Welborn TA, Knuiman MW (1992). Onset of NIDDM occurs at least 4-7 year before clinical diagnosis. Diabet Care.

[7] Groop L (2000). Pathogenesis of type 2 diabetes: the relative contribution of insulin resistance and impaired insulin secretion. Int J Clin Pract Suppl.

[8] Reaven GM (1988). Banting lecture 1988. Role of insulin resistance in human disease. Diabetes.

[9] Pyörälä M, Miettinen H, Laakso M, Pyörälä K Hyperinsulinemia predicts coronary heart disease risk in healthy middle-aged men: The 22-year follow-up results of the Helsinki Policemen Study. Circulation 1998.

[10] König D, Hörmann J, Predel HG, Berg A (2018). A 12-month lifestyle intervention program improves body composition and reduces the prevalence of prediabetes in obese patients. Obes Facts.

[11] Misra A, Gopalan H, Jayawardena R (2019). Diabetes in developing countries. J Diabetes.

[12] Kim NH, Kim JH, Kim YJ (2014). Clinical and metabolic factors associated with development and regression of nonalcoholic fatty liver disease in non-obese individuals. Liver Int.

[13] DeFronzo RA, Tobin JD, Andres R (1979). Glucose clamp technique: A method for quantifying insulin secretion and resistance. Am J Physiol.

[14] Boucher J, Kleinridders A, Kahn CR (2014). Insulin receptor signaling in normal and insulin-resistant states. Cold Spring Harb Perspect Biol.

[15] de Ferranti SD, de Boer IH, Fonseca V (2014). Type 1 diabetes mellitus and cardiovascular disease. Circulation.

[16] Rodrigues TC, Canani LH, Gross JL (2010). Metabolic syndrome, insulin resistance and cardiovascular disease in type-1 diabetes mellitus. Arq Bras Cardiol.

[17] Song Y, Manson JE, Tinker L (2007). Insulin sensitivity and insulin secretion determined by homeostasis model assessment and risk of diabetes in a multiethnic cohort of women: the Women's Health Initiative Observational Study. Diabetes Care.

[18] Hirai FE, Moss SE, Klein BE, Klein R (2008). Relationship of glycemic control, exogenous insulin, and C-peptide levels to ischemic heart disease mortality over a 16-year period in people with older-onset diabetes: the Wisconsin Epidemiologic Study of Diabetic Retinopathy (WESDR). Diabetes Care.

[19] de Rooij SR, Dekker JM, Kozakova M (2009). Fasting insulin has a stronger association with an adverse cardiometabolic risk profile than insulin resistance: the RISC study. Eur J Endocrinol.

[20] Lu YK, Dong J, Li YL (2022). Association between insulin resistance and incidence of carotid atherosclerotic plaque: A cohort study. Nutr Metab Cardiovasc Dis.

[21] Mossmann M, Wainstein MV, Gonçalves SC (2015). HOMA-IR is associated with significant angiographic coronary artery disease in non-diabetic, non-obese individuals: a cross-sectional study. Diabetol Metab Syndr.

[22] Balkau B (2000). The DECODE Study: diabetes epidemiology—collaborative analysis of diagnostic criteria in Europe. Diabetes Metab.

[23] Lee KK, Fortmann SP, Fair JM (2009). Insulin resistance independently predicts the progression of coronary artery calcification. Am Heart J.

[24] Neumann FJ, souse –Uva M, Ahlsson A (2018). ESC/EACTS Guidelines on myocardial Revascularization. Eur Heart J.

[25] American Diabetes Association (2013). Diagnosis and classification of diabetes mellitus. Diabetes Care.

[26] Williams B, Mancia G, Spiering W (2018). 2018 ESC/ESH Guidelines for the management of arterial hypertension: The Task Force for the management of arterial hypertension of the European Society of Cardiology (ESC) and the European Society of Hypertension (ESH). Eur Heart J.

[27] Adams KF, Schatzkin A, Harris TB (2006). Overweight, obesity, and mortality in a large prospective cohort of persons 50 to 71 years old. N Engl J Med.

[28] Catapano AL, Graham I, De Backer G (2016). 2016 ESC/EAS Guidelines for the Management of Dyslipidaemias. Eur Heart J.

[29] Seldinger SI (1953). Catheter replacement of the needle in percutaneous arteriography; a new technique. Acta Radiol.

[30] Gensini GG (1983). A more meaningful scoring system for determining the severity of coronary heart disease. Am J Cardiol.

[31] Di Pino A, DeFronzo RA (2019). Insulin resistance and atherosclerosis: implications for insulin-sensitizing agents. Endocr Rev.

[32] Ormazabal V, Nair S, Elfeky O (2018). Association between insulin resistance and the development of cardiovascular disease. Cardiovasc Diabetol.

[33] Bertoluci MC, Quadros AS, Sarmento-Leite R (2010). Insulin resistance and triglyceride/hdlc index are strongly associated with coronary artery disease. Diabetol Metab Syndr.

[34] Hedblad B, Nilsson P, Engström G (2002). Insulin resistance in non-diabetic subjects is associated with increased incidence of myocardial infarction and death. Diabet Med.

[35] Åberg D, Åberg ND, Jood K (2019). Homeostasis model assessment of insulin resistance and outcome of ischemic stroke in non-diabetic patients-a prospective observational study. BMC Neurol.

[36] Os I, Os A, Abdelnoor M (2005). Insulin sensitivity in women with coronary heart disease during hormone replacement therapy. J Women Health.

[37] Kim J, Chae YK, Chernoff A (2013). The risk for coronary heart disease according to insulin resistance with and without type 2 diabetes. Endocr Res.

[38] Fernández-Hernando C, Ackah E, Yu J (2007). Loss of Akt1 leads to severe atherosclerosis and occlusive coronary artery disease. Cell Metab.

[39] Karrowni W, Li Y, Jones PG (2013). Insulin resistance is associated with significant clinical atherosclerosis in nondiabetic patients with acute myocardial infarction. Arterioscler Thromb Vasc Biol.

[40] Srinivasan MP, Kamath PK, Manjrekar PA (2013). Correlation of severity of coronary artery disease with insulin resistance. N Am J Med Sci.

[41] de Lima Sanches P, de Mello MT, Elias N (2020). Insulin resistance predicts severity of coronary atherosclerotic disease in non-diabetic patients. J Clin Med.

[42] de Lima Sanches P, de Mello MT, Elias N (2011). Improvement in HOMA- IR is an independent predictor of reduced carotid intima –media thickness in obese adolescents participating in an interdisciplinary weight loss program. Hypertens Res.

[43] Vonbank A, Saely CH, Rein P (2011). Insulin resistance is associated with the metabolic syndrome and is not directly linked to coronary artery disease. Clin Chim Acta.

[44] Solymoss BC, Marcil M, Chaour M (1995). Fasting hyperinsulinism, insulin resistance syndrome, and coronary artery disease in men and women. Am J Cardiol.

[45] El-Zanaty F, Way A (2009). Egypt demographic and health survey 2008. Cairo, Egypt. Ministry of Health, El-Zanaty and Associates, and Marco International.

[46] Raafat N, Abbas MA, Salem HM (2012). Malathion exposure and insulin resistance among a group of farmers in Al- Sharkia goveronate. Clin Biochem.

